# Supplementary material for: Reducing sialylation in melanoma increases classical pathway-mediated complement activation
Source: iScience. 2026 Aug 25;29(9):117326. doi: 10.1016/j.isci.2026.117326 (PMC13542485; doi:10.1016/j.isci.2026.117326)
Supplement: Supplementary file 1 [file mmc1.pdf]

iScience, Volume *29*

Supplemental information

Reducing sialylation in melanoma increases classical pathway-mediated complement activation

Magali Coccimiglio, Georgia Clayton, Elisa C. Toffoli, Tanja D. de Gruijl, Richard B. Pouw, Fabrizio Chiodo, and Yvette van Kooyk

Supplementary Figures

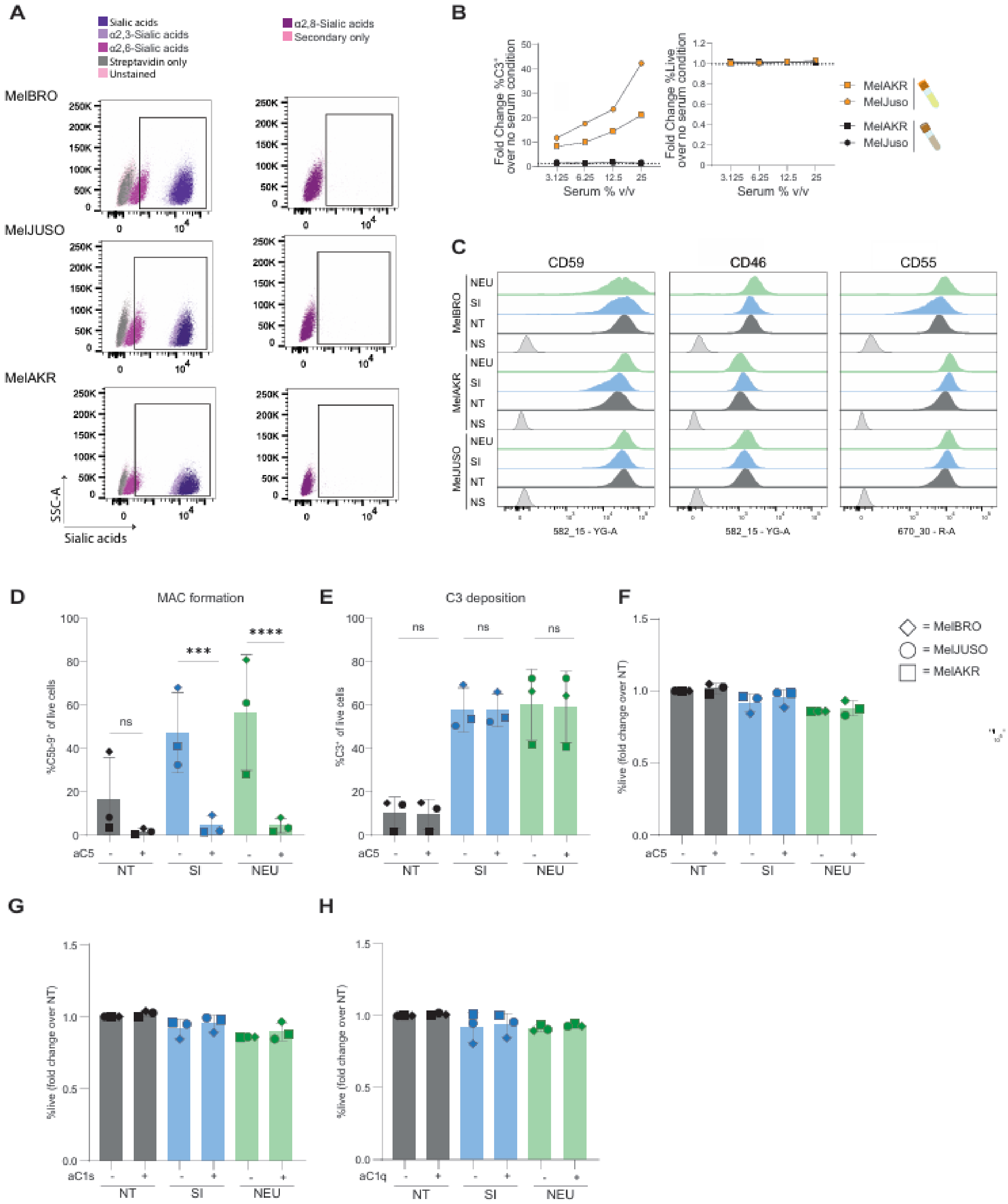

Supplementary Figure S1. Tumor sialylation prevents C3 deposition and MAC formation. (A) Dot plots per cell line associated to data form Figure 1A and B, showing in the left the expression of all sialic acids, α2,3- and α2,6-sialic acids with their negative control (streptavidin only) and unstained cells, and on the right expression of α2,8-sialic acids with the negative control (secondary only). **(B)** C3 deposition (left) and viability (right) determined by flow cytometry staining with Fixable Viability Dye eFluor450 of melanoma cells after 1 hour incubation with increasing amounts of serum or inactivated

serum as described in Fig.1C. Data shown as fold change over the no serum condition. **(C)** Histograms showing the expression of complement regulators CD59, CD46 and CD55 on melanoma cell lines non-treated (NT) or after treatment with sialyltransferases inhibitor (SI) or neuraminidase (NEU). NS = unstained control. **(D,E)** MAC formation (D) and C3 deposition (E) on NT, SI- or NEU-treated melanoma cells after 1 hour incubation with 25% (v/v) serum in the presence or absence of eculizumab (aC5). Data shown as mean ± s.d. from three independent experiments. **(F-H)** Percentage of live cells from single cells determined by flow cytometry staining with Fixable Viability Dye eFluor450 on NT, SI- or NEU-treated melanoma cells after 1 hour incubation with 25% (v/v) serum in the presence or absence of eculizumab (aC5) (F), aC1s (G) or aC1q (H). Data shown as mean ± s.d. from three independent experiments. Nested one-way ANOVA with Tukey's multiple comparisons test used for statistical analysis. ns = $p_{adj}>0.1$, * = $p_{adj} < 0.05$, ** = $p_{adj} < 0.01$, *** = $p_{adj} < 0.001$, **** = $p_{adj} < 0.0001$.

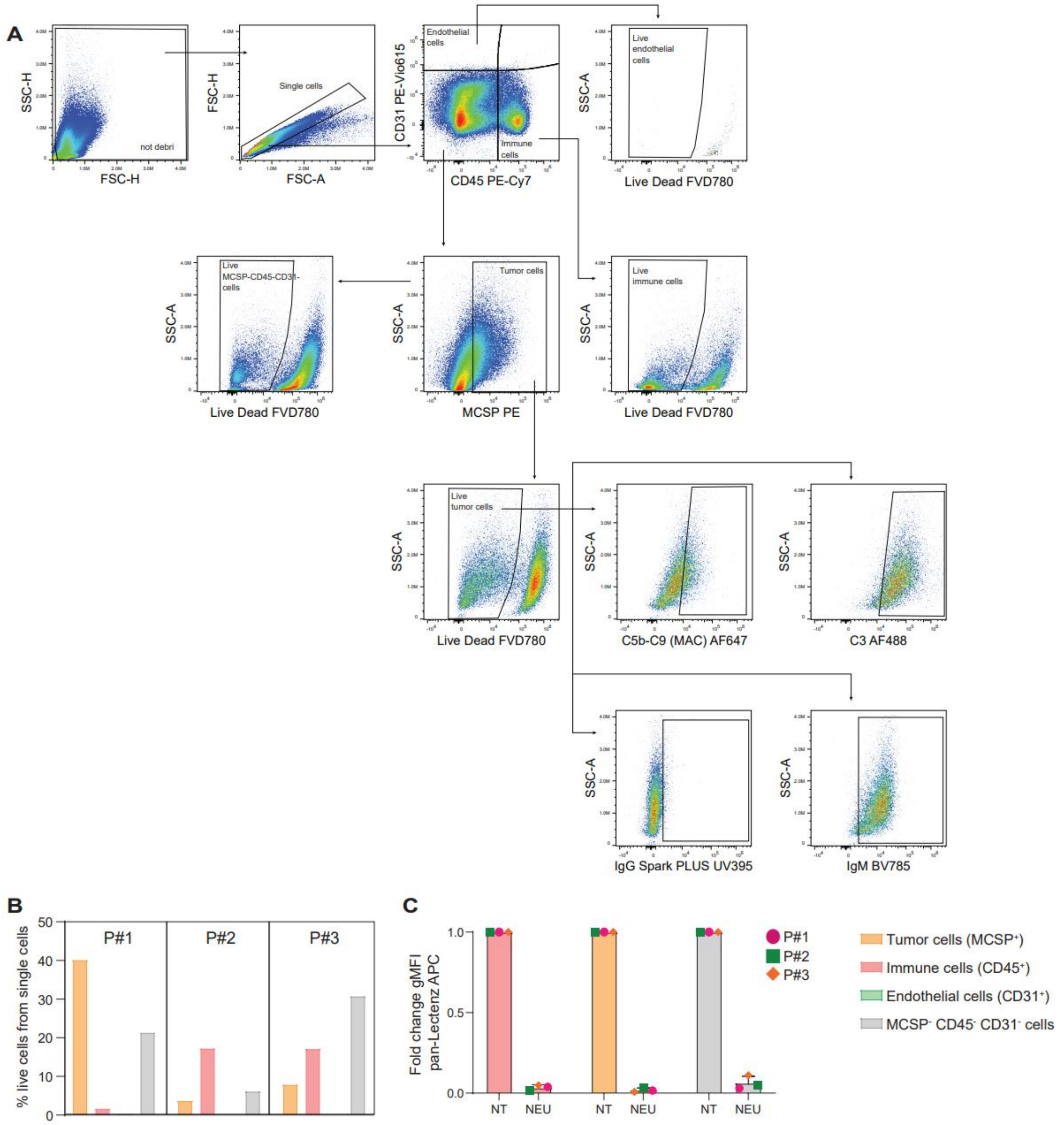

Supplementary Figure S2. Targeting sialylation in melanoma patient samples promotes complement activation on tumor cells through the classical pathway. (A) Gating strategy used for spectral flow cytometry panel on patient samples. The gates were set according to Fluorescence Minus One (FMO) controls and the no serum condition. **(B)** Frequency of tumor cells, immune cells, endothelial cells and the rest of cells in each patient sample. **(C)** Sialic acids expression on cells form patient samples after treatment with NEU. Data shown as fold change geometric mean fluorescence intensity (gMFI) over the NT condition. P#= patient number.
